# Glucose-6-Phosphate dehydrogenase deficiency associated hemolysis in a cohort of new onset type 1 diabetes children in Guangdong province, China

**DOI:** 10.1186/s13098-022-00812-1

**Published:** 2022-03-21

**Authors:** Aijing Xu, Minyan Jiang, Wen Zhang, Yunting Lin, Yongxian Shao, Huifen Mei, Jing Cheng, Cuili Liang, Cuiling Li, Xiuzhen Li, Li Liu

**Affiliations:** Department of Genetics and Endocrinology, Guangzhou Women and Children’s Medical Center, Guangzhou Medical University, 9 Jinsui Road, Guangzhou, 510623 China

**Keywords:** Glucose-6-phosphate dehydrogenase deficiency, Hemolysis, Type 1 diabetes

## Abstract

**Background:**

Glucose-6-phosphate dehydrogenase (G6PD) deficiency is one of the most common human genetic abnormalities, with a high prevalence in Guangdong, China. The purpose of this study was to explore the characteristics of newly diagnosed type 1 diabetes (T1D) patients with G6PD deficiency in a cohort of Chinese children and to investigate the relationship between the diabetic ketoacidosis (DKA) and hemolysis due to G6PD deficiency in these patients.

**Methods:**

A total of 503 newly diagnosed T1D children aged 6 months–18 years were collected and their G6PD enzyme activity were measured. Fasting plasma glucose (FPG), hemoglobin A1c (HbA1c), and *G6PD* gene were analysed. The pH, HCO_3_, and plasma osmotic pressure between DKA patients with and without hemolysis at the presentation were compared.

**Results:**

In the present study, G6PD deficiency accounted for 5.3% of newly diagnosed T1D children. There were no significant differences in FPG/HbA1c and HbA1c levels between T1D children alone and T1D children with G6PD deficiency. Hemolysis appeared in five of the twenty-two DKA patients with G6PD deficiency. Two patients had fever at onset and were given ibuprofen and cefazolin. The other three patients did not have infection or ingestion of hemolytic drugs. There were no significant difference in pH, HCO_3_, and osmotic pressure between the children with DKA with and without hemolysis at the presentation. The hemolysis occurred between 2 and 7 days after admission and the hyperglycaemia had been corrected by the time hemolysis occurs. Four *G6PD* gene mutations were found in the diabetes with G6PD deficiency patients: c.1376G > T, c.1388G > A, c.95A > G, and c.871G > A, all of which were genes with high frequency of G6PD deficiency in Guangdong Province. No correlation between genotype and hemolysis was found.

**Conclusion:**

In the present study, we found the frequency of G6PD deficiency among newly diagnosed T1D children was similar to that of the general population. However, DKA children with G6PD deficiency are prone to occur hemolytic anemia, and these hemolysis usually occurs when DKA is corrected and blood glucose is in homeostatic state, which is easy to be ignored. To reduce the risk of this complication, especially in areas with high incidence of G6PD deficiency, screening for G6PD activity in people with newly diagnosed diabetes should be considered.

## Background

Glucose-6-phosphate dehydrogenase (G6PD) deficiency is an X-linked hereditary genetic defect caused by mutations in the *G6PD* gene, and is the most common enzyme deficiency worldwide. The highest prevalence is reported in tropical and subtropical regions, including Southern China [1]. Recent study showed that the prevalence of G6PD deficiency in Guangdong, China was 3.4% which was higher than the national level 2.1% [2]. G6PD is an enzyme that catalyzes the first step in the pentose phosphate pathway. G6PD deficiency reduced nicotinamide adenine dinucleotide phosphate (NAPDH) which is required for protection of red blood cells from oxidative stress [3]. The most common clinical manifestations of G6PD deficiency are acute hemolysis, hyperbilirubinemia, and chronic hemolysis. The acute hemolysis is usually triggered by oxidative injuries with G6PD deficiency including certain food, medications, infections, and rarely diabetic ketoacidosis [4]. Only a few reports of hemolysis induced by G6PD deficiency in patients with type 1 diabetes mellitus (T1D) have been described previously, especially in children. Other reports also described the G6PD deficiency trigger hemolysis in diabetic ketoacidosis (DKA) [5]. Whereas, the correlation between G6PD deficiency hemolysis and DKA remains controversial [6]. G6PD deficiency hemolysis among diabetic subjects may be related to ketoacidosis [7], blood glucose normalisation [8], hypoglycaemia [9] and administration of metformin [10] or glibenclamide [11].

In the present study, we explored the characteristics of newly diagnosed T1D patients with G6PD deficiency and reported the patients in DKA developed hemolysis due to G6PD deficiency.

## Materials and methods

### Subjects

There were 503 children with newly diagnosed type 1 diabetes between Septemper 2015 and April 2021 at Guangzhou Women and Children’s Medical Center, Guangzhou, China. All patients were aged 6 months– 18 years. The clinical diagnosis of T1D and DKA was according to the 2018 International Society of Pediatric and Adolescent Diabetes (ISPAD) Clinical Practice Consensus Guidelines [12, 13]. The clinical manifestations of DKA include: (1) Dehydration; (2) Tachypnea; deep, sighing (Kussmaul) respiration; breath has the smell of acetone; (3) Nausea, vomiting,and abdominal pain; (4) Confusion, drowsiness, progressive obtundation and loss of consciousness [13]. DKA was treated according to previously described guidelines [13]. The criteria for clinical diagnosis of acute hemolysis was when the child presented with sudden jaundice, pallor and had a dark or cola-colored urine, an elevated reticulocyte count, indirect bilirubin, and lactate dehydrogenase.

The study was performed according to the Declaration of Helsinki. The Ethics Committee of the Guangzhou Women and Children’s Medical Center approved the study (Code of Ethics: 2020–49800). Written informed consent was obtained from the patients’ parents.

### Laboratory evaluation

The following basic clinical laboratory parameters were measured by each subject: routine blood tests, urine analysis, alanine aminotransferase (ALT) level, lactic dehydrogenase (LDH) level, arterial blood gases, electrolytes, and serum bilirubin were determined using standard techniques. For these T1D patients with G6PD deficiency, a routine blood test was conducted once a day for the first 3 days after admission; If hemoglobin was normal, the routine blood tests were performed every 3 days until discharge and once a week for 2 weeks after discharge; If hemoglobin decreased, such tests conducted every 2–3 days until hemoglobin returns to 85 g/L, and then tested it every 3–5 days until hemoglobin returns to normal. Arterial blood gas analysis was detected on admission, and repeated 2 to 4 hourly when DKA occurred. FPG was determined by the enzymatic method; HbA1c was measured by latex immunoagglutination inhibition methodology (DCA Systems; Siemens, Erlangen, Germany).

### Measurement of erythrocyte G6PD enzyme activity

Enzyme activity was assayed by a commercial G6PD Detection Assay Kit (KOFA Medical, Guangzhou, China), according to the method suggested by WHO for measuring the G6PD/6PGD (6-phosphogluconate dehydrogenase) ratio [14]. The tests were performed according to the manufacturer’s instructions. The normal range of G6PD/6PGD ratio for children was locally defined as 1.0–2.3.

### G6PD gene analyses

Genomic DNA was extracted from blood samples using a QIAamp DNA Blood Mini kit (Qiagen, Hilden, Germany) according to the manufacturer’s instructions. The entire coding exons and exon–intron boundaries of the *G6PD* gene were amplified by polymerase chain reaction (PCR) using specific primers. The PCR products were directly sequenced on the ABI 3730xl Genetic Analyzer (Applied Biosystems, Foster City, CA, USA). The sequencing chromatograms were analyzed by comparing with the reference sequences (NM_001042351) using DNAMAN software (Lynnon Biosoft, Inc., Ouebec, Canada).

### Statistical analysis

All statistical analyses were performed with SPSS version 17.0 (International Business Machines Corporation, Armonk, NY, USA). All results are presented as the mean ± standard deviation (SD) and as percentages (%). Group comparison was performed by Student's t-test or Chi-square test. *P* < 0.05 was considered statistically significant.

## Results

In the present study, we obtained and analyzed data of 503 children and adolescents with newly diagnosed T1D from Septemper 2015 to April 2021, 27 (27/503, 5.3%) with G6PD deficiency. Among these T1D with G6PD deficiency patients, 6 females and 21 males, the mean age of these subjects at the time of diagnosis of diabetes was 8.0 ± 5.1 years. Twenty two patients were combined with DKA and five of them showed acute hemolysis. There was no evidence of hemolysis in the remaining 17 episodes of DKA. Baseline characteristics were listed in Table 1. There were no significant differences in clinical characteristics and HbA1c level between the G6PD deficiency diabetes group and G6PD non-deficiency diabetes group.

**Table 1:** Baseline characteristics of the newly diagnosed type 1 diabetes patients with or without G6PD deficiency on admission

	G6PD non-deficiency (n = 29)	G6PD deficiency (n = 27)	*t* value	*P* value
Age (y)	7.6 ± 3.5	8.0 ± 5.1	0.17	0.86
Gender (female/male)	13/16	6/21		
DKA (%)	23 (79%)	22 (81%)	χ^2^ = 0.04	0.84
HbA1c (%)	12.13 ± 1.61	11.28 ± 2.48	0.91	0.37
FPG/HbA1c	2.25 ± 0.86	2.35 ± 0.75	0.36	0.72
HGB (g/L)	126.12 ± 8.72	119.90 ± 17.30	1.15	0.26
G6PD/6PGD	1.37 ± 0.16	0.36 ± 0.34	9.45	0.00
*G6PD* gene mutations		c.1388G > A (p.Arg493His) (n = 6) c.1376G > T (p.Arg459Leu) (n = 7) c.95A > G (p.His32Arg) (n = 4) c.871G > A (p.Val291Met) (n = 1)		

*DKA* diabetic ketoacidosis, *HbA1c* hemoglobin A1c, *FPG* fasting plasma glucose, *HGB* hemoglobin, *G6PD* glucose-6-phosphate dehydrogenase, *6PGD* 6-phosphogluconate dehydrogenase

**Fig. 1:**
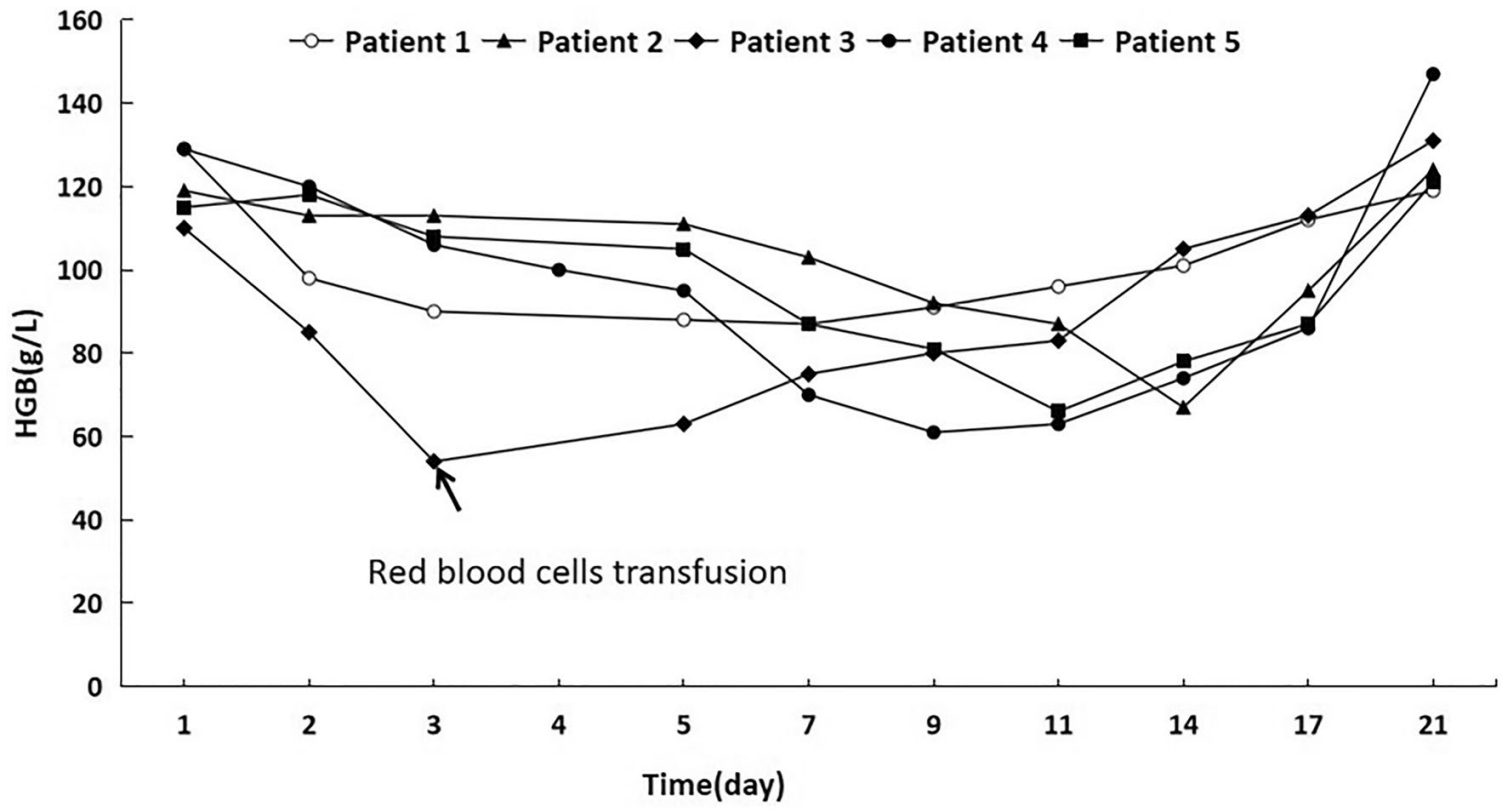
Changes in HGB levels during hemolysis in glucose-6-phosphate dehydrogenase deficiency with diabetic ketoacidosis children

The acute hemolysis was occurred in five DKA patients. There were four males and one female, aged form 1 year and 8 months to 13 years. None of these patients had suffered a hemolytic episode in the past. Two patients (patient 2 and 3) had a fever at the time of DKA onset, with a temperature beyond 38.5 ℃, and took ibuprofen and cefazolin, then the hemolysis occurred at 7 days and 2 days after admission, respectively. While, the other three patients had no infections and no drugs ingested, and the hemolysis occurred 2, 4, 7 days after admission, respectively. We compared the pH, HCO_3_, plasma osmolality, and G6PD activity of the patients with DKA on admission between hemolysis and non-hemolysis groups (Table 2). The FPG levels and HbA1c of the hemolysis group were higher than non-hemolysis group, while G6PD activity of the hemolysis group was lower than non-hemolysis group, but no significant difference between the two groups. The clinical and laboratory results of the patients at the time of hemolysis were shown in Table 3. When the hemolysis occurred, the metabolic balance of these children was improved and the glycemic levels decreased (Table 3). The haemoglobin levels during hemolysis were shown in the Fig. 1. For patient 3, the hemolytic episode was not self-remission and he needed a red blood cells transfusion. Furthermore, he developed DKA again 8 months later, but hemolysis did not occur again.

**Table 2:** Baseline characteristics of the G6PD deficiency complicated with diabetic ketoacidosis with or without hemolysis on admission

	Non-hemolysis (n = 17)	Hemolysis (n = 5)	*t* value	*P* value
Age (y)	7.8 ± 5.2	5.9 ± 4.9	0.75	0.46
Gender (female/male)	4/13	1/4		
HbA1c (%)	11.05 ± 2.47	12.11 ± 1.73	0.80	0.43
FPG (mmol/L)	24.95 ± 7.26	29.55 ± 1.91	1.24	0.21
HGB (g/L)	121.72 ± 13.91	122.30 ± 8.31	0.08	0.94
G6PD/6GPD	0.35 ± 0.34	0.13 ± 0.07	1.41	0.17
pH	7.16 ± 0.11	7.15 ± 0.12	0.22	0.84
HCO_3_ (mmol/L)	8.98 ± 4.75	6.42 ± 5.05	0.84	0.42
Plasma osmolality (mOsm/kg)	287.30 ± 8.55	289.01 ± 5.09	0.37	0.72

*FPG* fasting plasma glucose, *HGB* hemoglobin, *G6PD* glucose-6-phosphate dehydrogenase, *6PGD* 6-phosphogluconate dehydrogenase

**Table 3:** Clinical and laboratory tests of 5 diabetic ketoacidosis with hemolysis children at the time of hemolysis occurs

Patient	1	2	3	4	5	Normal range
Age at onset	9y	2y8m	3y	13y	1y8m	
Gender	F	M	M	M	M	
Symptoms	Pallor	Pallor	Cola-colored urine	Jaundice	Cola-colored urine	
Hemoloysis occurs after admission(d)	2	7	2	4	7	
HGB (g/L)	98	103	85	100	87	105–145
Ret (%)	6.8	10.9	6.2	6.1	4.5	0.5–1.5
WBC (× 10^9^/L)	5.9	6.2	8.3	8.3	17.0	5–12
CRP (mg/L)	1.25	2.21	1.91	1.29	0.08	< 8.2
FPG (mmol/L)	7.2	13.3	11.2	9.4	8.7	3.9–6.1
TBIL (umol/L)	24.2	40.0	20.8	130.5	34.2	2–17
IBIL (umol/L)	18.8	31.0	16	124.5	25.5	2–13.7
ALT (U/L)	16	20	16	21	28	9–50
LDH (U/L)	336	357	420	178	213	159–322
BUN (mmol/L)	2.9	2.8	2.5	3.1	4.1	2.7–7.1
Coombs test	–	–	–	–	–	–
Urobilinogen	+	+	+	+ +	+ +	–
pH	7.43	7.45	7.35	7.33	7.4	7.31–7.41
HCO_3_ (mmol/L)	22	22.5	20.2	22.9	22.3	18.6–22.6
Plasma osmolality (mOsm/kg)	293	285	295	290	291	285–295
G6PD/6PGD	0.12	0.10	0.12	0.26	0.06	1.0–2.3
*G6PD* gene	c.1388G > A	c.1376G > T	c.95A > G	Not detected	c.1388G > A	

*HGB* hemoglobin, *Ret* reticulocyte, *WBC* white blood cells count, *CRP* c-reactive protein, *FPG* fasting plasma glucose, *TBIL* total bilirubin, *IBIL* indirect bilirubin, *ALT* alanine aminotransferase, *LDH* lactate dehydrogenase, *BUN* blood urea nitrogen, *G6PD* glucose-6-phosphate dehydrogenase, *6PGD* 6-phosphogluconate dehydrogenase

*G6PD* gene mutations were detected in 18 of the 27 T1D patients with G6PD deficiency. Four kinds of *G6PD* variants were identified in our study. The characteristics of *G6PD* genotype observed from our samples were listed in Table 4. The *G6PD* variants were *G6PD* Canton (1376 G > T), *G6PD* Kaiping (1388 G > A), *G6PD* Gaohe (95 A > G), and Viangchan (871G > A), the most common variants in the Guangdong population of China. Five of the six female G6PD deficiency patients underwent genetic testing, and three patients were 1388 G > A homozygous mutation, one was 1376 G > T homozygous mutation, and one was 1376 G > T heterozygous mutation. Three *G6PD* gene variants were found in four hemolysis patients: c.1376G > T, c.1388G > A and c.95A > G.

**Table 4:** Pathogenic mutations and their descriptions of 18 type 1 diabetes with G6PD deficiency patients

Patient	Age at onset	Gender	*G6PD* gene					G6PD/6PGD
			Mutation name	Exon	Nucleotide change	Amino acid change	Genotype	
1	9y	F	Kaiping	12	c.1388G > A	p.Arg493His	Homo	0.12
2	2y8m	M	Canton	11	c.1376G > T	p.Arg459Leu	Hemi	0.10
3	3y	M	Gaohe	2	c.95A > G	p.His32Arg	Hemi	0.12
5	1y8m	M	Kaiping	12	c.1388G > A	p.Arg493His	Hemi	0.06
6	12y	M	Canton	11	c.1376G > T	p.Arg459Leu	Hemi	0.09
7	11y	M	Kaiping	12	c.1388G > A	p.Arg493His	Hemi	0.12
8	16y	M	Gaohe	2	c.95A > G	p.His32Arg	Hemi	0.12
9	10 m	M	Gaohe	2	c.95A > G	p.His32Arg	Hemi	0.88
10	2y3m	M	Gaohe	2	c.95A > G	p.His32Arg	Hemi	0.06
11	3y	F	Kaiping	12	c.1388G > A	p.Arg493His	Homo	0.26
12	13y10m	F	Kaiping	12	c.1388G > A	p.Arg493His	Homo	0.34
13	7y	F	Canton	11	c.1376G > T	p.Arg459Leu	Hetero	0.94
14	1y2m	M	Kaiping	12	c.1388G > A	p.Arg493His	Hemi	0.09
15	7y	M	Canton	11	c.1376G > T	p.Arg459Leu	Hemi	0.39
16	7y	M	Canton	11	c.1376G > T	p.Arg459Leu	Hemi	0.09
17	5y	M	Viangchan	9	c.871G > A	p.Val291Met	Hemi	0.77
18	9y	F	Canton	11	c.1376G > T	p.Arg459Leu	Homo	0.28
19	11 m	M	Canton	11	c.1376G > T	p.Arg459Leu	Hemi	0.16

*Hemi* hemizyotes, *Homo* homozygous, *Hetero* heterozygous

## Discussion

We describe here a series of newly diagnosed T1D children with G6PD deficiency. DKA occurs in approximately 81% of these patients, hemolysis induced by G6PD deficiency in five patients with DKA.

The prevalence of G6PD deficiency is highly variable around the world, even in different regions of the same country. The highest prevalence is reported in Africa, Southern Europe, the Middle East, Mediterranean countries, Southeast Asia, including Southern China. It has been reported as occurring with a varying incidence of from 3.1 to 16.1% in Guangdong [14, 15]. The relationship between G6PD deficiency and diabetes had been studied. Several studies have shown that a higher prevalence of G6PD deficiency among patients with diabetes mellitus compared to healthy population control [16–18]. While, other studies reported that there was no difference in the prevalence of G6PD deficiency among diabetics and control [19, 20]. In the present study, we found a 5.1% frequency of G6PD deficiency among T1D patients, which was simillar to that of the general population.

A reduced erythrocyte lifespan can cause falsely lower HbA1c levels, resulting in underestimation of HbA1c in diabetes mellitus patients [21]. Chang et al. [22] reported that diabetes mellitus patients with G6PD deficiency had significantly higher FPG/HbA1c ratios than those without G6PD deficiency. Wheeler et al.[21] estimated that about 650,000 African Americans with type 2 diabetes (T2D) would be missed if screened with HbA1c alone because of the G6PD variant lowered HbA1c levels. While, Heymann [16] reported that there was no significant difference in HbA1c levels between patients with or without G6PD deficiency. We also compared the FPG/HbA1c ratios between T1D children with and without G6PD deficiency, but we did not find a significant difference between these two groups. At the same time, there was no significant difference in HbA1c levels between these two groups.

Hemolysis in G6PD deficient individuals is usually associated with the ingestion of fava beans, certain drugs, metabolic conditions, and infections. Hemolytic anaemias in diabetes mellitus patients with G6PD deficiency are generally attributed to infections or administration of metformin [10] or glibenclamide [11]. However, the acute hemolysis due to G6PD deficiency in patients with DKA has been reported, but most of these are case reports, and the underlying mechanism remains poorly understood. Moreover, the correlation between G6PD deficiency hemolysis and DKA remains controversial.

In the present study, DKA occurred in 22 of the 27 T1D children with G6PD deficiency, and hemolysis occurred in five DKA patients. Two of these five patients had fever at the DKA onset and took the ibuprofen and antibiotics. The other three patients did not indicate infections, ingestion of specific food or drug administration that could be responsible for hemolysis. We also compared the pH, HCO_3_, and plasma osmotic pressure between the DKA with and without hemolysis at the presentation, and there was no significant difference. Then, hemolysis was not triggered by acid–base disequilibrium. DKA treatment according to ISPAD guidelines, insulin and potassium chloride were the drugs used for the treatment. The other possible causes of acute hemolysis and other oxidative stress that can cause hemolysis were also ruled out in these three patients. Moreover, the hemolysis of all five patients occurred at 2–7 days after admission, the acid–base imbalance of DKA was improved, and the glycemic levels was significantly lower than in the previous days. Thus, the supposed mechanisms of these cases may be the rapid correction of hyperglycemia, which was reported previously [23].

G6PD catalyzes the first step of the pentose phosphate pathway, converts glucose-6-phosphate into 6-phosphogluconolactone, producing reduced NADPH. NADPH is used to maintain the supply of reduced glutathione (GSH), which plays a significant role in regulating antioxidant balance and thus protecting cells against oxidative damage. Under physiological condition, the glycolytic pathway breaks down nearly 80–90% of the body’s glucose, while the pentose phosphate pathway consumes the remaining 10–20%. Under hyperglycemic condition, more glucose will flux through the glycolytic pathway that produces more NADH production [24]. Multiple signaling pathways can branch off the glycolytic pathway under chronic hyperglycemic conditions. A major pathway is the polyol pathway, in which NADPH is consumed and, when NADPH level decreased so does reduced form of glutathione (GSH) [24].These pathways are minor and insignificant under normoglycaemic condition. When treated with DKA, during insulin infusion, rapid correction of prolonged hyperglycaemia may increased the inability of the red blood cells to produce NADPH. This leads to an imbalance in the consumption and production of NADPH [25]. In addition, hyperglycaemia increased the production of reactive oxygen species (ROS) in the form of superoxide [26]. These reactive oxygen species can destroy and deform the erythrocyte membrane, which leads to erythrocytes to be fragile and prone to lysis [25]. So, it is important to avoid the rapid decline in glucose availability.

The present study also evaluated the correlation between hemolysis and G6PD activity and *G6PD* gene mutations. We compared the G6PD activity between DKA with and without hemolysis, but there was no significant difference between the two groups. Sobngwi et al. [27] reported that G6PD deficiency is associated with the severity of pancreatic β-cell failure. The severe insulin deficiency and metabolic decompensation in Ketosis-prone diabetes can alter G6PD activity. In this study, we found four *G6PD* gene mutations in the 18 T1D with G6PD deficiency children: c.1376G > T, c.1388G > A, c.95A > G, and c.871G > A, the most frequent G6PD pathogenic variants in Guangdong [28]. Three *G6PD* gene mutations were found in 4 hemolysis patients: c.1376G > T, c.1388G > A and c.95A > G. In our study, DNA analysis did not reveal a relationship between hemolysis and *G6PD* gene mutations.

In the present cases, only 5 of the 22 DKA patients with G6PD deficiency developed hemolysis. For patient 3, DKA episodes occurred twice, but only at the new onset of T1D with DKA developed hemolysis. When he had DKA for the second time, the hyperglycemia was reduced slowly, and he didn't have hemolysis again. The hemolysis in these individuals occurred between 2 to 7 days after normalisation of hyperglycaemia in newly diagnosed diabetes, which is consistent with that previously reported between 1 to 14 days [24]. Furthermore, severe hemolysis had been reported after the patient discharged [29]. Hence, for T1D with G6PD deficiency patients, we recommend careful clinical surveillance in these patients even correction of the hyperglycemia.

The limitation of this study was the relatively small sample size. The hemolysis caused by DKA is still unclear. Therefore, further investigation using a larger sample size and further cytological experiments are needed to confirm the present findings and explore the potential mechanisms of G6PD hemolysis with T1D.

## Conclusions

In the present study, we found the frequency of G6PD deficiency among newly diagnosed T1D children was similar to that of the general population.There was no significant difference in FPG/HbA1c ratio or HbA1c levels between T1D children with and without G6PD deficiency. The acute hemolysis due to G6PD deficiency may be one of the rare acute complications of new onset diabetes. Due to the hemolysis was a result of the rapid correction of hyperglycaemia, it is important to treat hyperglycemia less aggressive. To reduce the risk of hemolysis, screening for G6PD activity in people with newly diagnosed diabetes in areas with high incidence of G6PD deficiency should be considered.

## Data Availability

The datasets used and/or analysed during the current study are available from the corresponding author on reasonable request.
